# Amino acid supplementation during the adaptation period did not affect the standardized ileal digestibility of amino acids in corn and soybean meal fed to pigs

**DOI:** 10.5713/ab.23.0331

**Published:** 2023-11-02

**Authors:** Hyunjun Choi, Sun Jong You, Beob Gyun Kim

**Affiliations:** 1 Department of Animal Science and Technology, Konkuk University, Seoul 05029, Korea

**Keywords:** Amino Acid Mixture, Amino Acids, Ileal Digestibility, Swine

## Abstract

**Objective:**

The objective was to determine the influence of amino acid (AA) supplementation during the adaptation period on the ileal digestibility of crude protein and AA in corn and soybean meal (SBM) fed to pigs.

**Methods:**

Six barrows with an initial body weight of 30.9±2.6 kg fitted with a T-cannula at the distal ileum were assigned to a 6×6 Latin square design with 6 dietary treatments and 6 periods. Two experimental diets contained corn or SBM as the sole AA source and an N-free diet was additionally prepared. For AA supplementation groups, an AA mixture consisted of Gly, Lys, Met, Thr, Trp, Ile, Val, His, and Phe was added to the corn diet and the N-free diet at the expense of cornstarch, and an AA mixture of Lys, Met, and Thr was added to the SBM diet. All diets contained 0.5% of chromic oxide. The 6 experimental diets were fed to the pigs for four and half days, and the 3 diets containing an AA mixture were switched to the respective diets without AA mixture during the following two and half days. Ileal digesta were collected on days 6 and 7.

**Results:**

The addition of an AA mixture during the adaptation period increased apparent ileal digestibility of Arg and Trp in corn (p<0.05) but did not affect that in SBM. The addition of an AA mixture during the adaptation period increased apparent ileal digestibility of Pro and Gly regardless of feed ingredient (p<0.05) but did not affect that of other AA. All AA except Pro in corn and SBM were unaffected by the addition of the AA mixture during the adaptation period.

**Conclusion:**

The addition of amino acids to a low-protein diet during the adaptation period does not affect the standardized ileal digestibility of indispensable amino acids in pigs.

## INTRODUCTION

A supply of proper quantity of amino acids (AA) is essential for the maintenance and maximal growth of pigs [1]. The requirements of AA for pigs and the AA concentrations in feed ingredients have been suggested as total AA and ileal digestible AA [1,2]. Ileal digestible AA are more widely used for the formulation of swine diets to reflect biological availability of AA because microbiota in the hindgut change the AA composition [3]. The use of ileal digestible AA rather than total AA in pig diet formulations has been shown to improve nitrogen (N) utilization [4]. To determine ileal AA digestibility in a feed ingredient of interest, the ingredient is included in the experimental diet as the sole source of AA. Thus, AA compositions in experimental diets are often insufficient or imbalanced for maintaining a normal physiological condition of pigs, particularly in an N-free diet and cereal grain-based diets [5].

The AA deficiency in the experimental diets has been often neglected in ileal AA digestibility experiments, assuming no major effects of AA deficiency on ileal AA digestibility [6–8]. In other experiments, however, crystalline AA have been supplemented during the 5 days of adaptation period before 2 days of ileal digesta collection from pigs [7,9] mainly to lower potential influences of AA deficiency on ileal AA digestibility. However, the AA mixture supplementation during adaptation period did not influence the ileal digestibility of crude protein (CP) and AA in corn and soybean meal (SBM) of growing pigs [10]. To best our knowledge, the information on the effects of AA mixture supplementation during adaptation period on basal endogenous losses (BEL) of CP and AA and ileal digestibility of CP and AA in corn and SBM of growing pigs is very limited. Therefore, the present experiment was conducted to determine the effects of AA mixture supplementation during the adaptation period on the BEL of AA and ileal digestibility of CP and AA in corn and SBM of growing pigs. The influence of AA supplementation during adaptation period of ileal digestibility experiments was hypothesized to differ depending on the AA composition of the diets.

## MATERIALS AND METHODS

### Animal care

The experimental procedure was approved by the Institutional Animal Care and Use Committee at Konkuk University (KU 18145).

### Animals, diets, and experimental design

Six barrows with an initial body weight of 30.9±2.6 kg fitted with a T-cannula at the distal ileum were assigned to a 6×6 Latin square design with 6 dietary treatments and 6 periods. The same batches of corn and SBM were used to formulate experimental diets (Table 1). Two experimental diets contained corn or SBM as the sole source of AA and an N-free diet was additionally prepared (Table 2). For AA supplementation groups, an AA mixture consisted of Gly, Lys, Met, Thr, Trp, Ile, Val, His, and Phe (Table 3) based on the study by Pedersen et al [9] was added to the corn diet and the N-free diet at the expense of cornstarch, and an AA mixture of Lys, Met, and Thr was added to the SBM diet. All diets contained 0.5% chromic oxide as an indigestible index. Vitamin and trace minerals were supplemented to meet or exceed the requirement estimates of the NRC [1]. All diets were prepared in mash form using a flat-bottom vertical mixer [11]. Pigs were individually housed in pens (1.0 m×1.2 m^2^) that were equipped with a feeder and a nipple drinker. A spreadsheet program was used to balance potential carryover effects [12].

### Feeding and sample collection

During the experimental period, pigs were fed their respective diets at a level of 3.0 times the estimated energy requirement for maintenance (i.e., 197 kcal metabolizable energy/kg body weight^0.60^) [1]. Feed allowance was divided into 2 equal meals that were provided at 0800 and 1700 h. Pigs had free access to water through a nipple drinker in each pen. The 6 experimental diets were fed to the pigs for four and half days, and the 3 diets containing an AA mixture were switched to the respective diets without AA mixture during the following two and half days (Table 4). After 5 days of adaptation period, ileal digesta were collected from 0900 to 1700 on days 6 and 7. A plastic sample bag with wire was fixed to the T-cannula for the ileal digesta collection. The bag was removed whenever the plastic bag was filled with digesta, or at least once every 30 min. Collected samples were immediately stored at −20°C to prevent bacterial degradation of AA [6].

### Chemical analyses

The frozen ileal digesta samples were lyophilized in a freeze drier. Ingredient and diet samples were analyzed for dry matter (DM; method 930.15), CP (method 990.03), and ether extract (method 920.39) as described in AOAC [13]. Diet samples were also analyzed for ash (method 942.05), calcium (method 978.02), phosphorus (method 946.06), neutral detergent fiber (method 2002.04), and acid detergent fiber (method 973.18) [13]. Amino acids concentrations of diets and ileal digesta samples were determined by ion-exchange chromatography with post-column derivatization with ninhydrin. Before analysis, samples were liberated from the protein by hydrolysis with 6 mol/L HCl at 110°C for 24 h (method 982.30) [13]. Methionine and cystine were analyzed as methionine sulfone and cysteic acid, respectively, after cold performic acid oxidation overnight before hydrolysis. Tryptophan was determined after NaOH hydrolysis for 22 h at 110°C. Chromium (Cr) concentrations of diets and ileal digesta samples were determined by the procedure described in Fenton and Fenton [14].

### Calculation and statistical analyses

The apparent ileal digestibility (AID), BEL, and standardized ileal digestibility (SID) of CP and AA were calculated based on equations as follows [15]:


$$\begin{array}{l} \text{AID }(\%)=[1-({\text{AA}}_{\text{i}}/{\text{AA}}_{\text{d}})\times ({\text{Cr}}_{\text{d}}/{\text{Cr}}_{\text{i}})]\times 100\\ \text{BEL }(\text{g}/\text{kg DMI})={\text{AA}}_{\text{i}}\times ({\text{Cr}}_{\text{d}}/{\text{Cr}}_{\text{i}})\\ \text{SID }(\%)=\text{AID}+[(\text{BEL}/{\text{AA}}_{\text{d}}]\times 100] \end{array}$$

where AA_d_ and AA_i_ represent the concentration of CP or AA (g/kg DM) in diets and ileal digesta, respectively. The concentrations of Cr (g/kg DM) in diets and ileal digesta represent Cr_d_ and Cr_i_, respectively. The BEL of CP and AA calculated for pigs fed the N-free diet added with a crystalline AA mixture during the adaptation period were used for the calculation of SID values of pigs fed diets added with a crystalline AA mixture during the adaptation period.

Data were analyzed using the MIXED procedure of SAS (SAS Inst. Inc., Cary, NC, USA). In the statistical model for digestibility values for CP and AA, the fixed variable was diet. In the model for testing the effects of AA addition on the BEL of CP and AA, the fixed variable was AA addition. In both models, animal and period were used as random variables. Least squares of means for each treatment were calculated. Orthogonal contrasts were conducted to test the effect of feed ingredient, AA addition, and the interaction between the effect of feed ingredient and AA addition. When the interaction between the effect of feed ingredients and AA addition is significant, a pairwise comparison was conducted using the least significant difference procedure with Tukey’s adjustment. The experimental unit was a pig, and the statistical significance was declared at an alpha level of 0.05.

## RESULTS

The analyzed AA contents in experimental diets were in good agreement with calculated values (Table 5). The addition of an AA mixture during the adaptation period increased (p< 0.05) AID of Arg and Trp in corn, but not in SBM (Table 6). The addition of an AA mixture during the adaptation period increased AID of CP, Pro, and Gly regardless of feed ingredients (p<0.05) but did not affect AID of other AA. The AID of all indispensable AA except Leu was greater (p<0.05) in SBM compared with corn. In pigs fed an N-free diet, the addition of an AA mixture during the adaptation period did not affect the BEL of CP and AA (Table 7).

No interaction between ingredient and AA mixture addition was observed in the SID of AA (Table 8). The addition of an AA mixture during the adaptation period did not affect the SID of AA except that the SID of CP and Pro was increased (p<0.05) by the addition of an AA mixture during the adaptation period. The SID of all indispensable AA except Leu, Met, Thr, and Val was greater (p<0.05) in SBM compared with corn.

## DISCUSSION

The chemical compositions in corn and SBM used in the current study were similar to the values in the previous studies [1,6]. The BEL of CP and AA in the present work were within a range reported in the previous studies [6,16,17]. The BEL of CP and AA secreted in the gastrointestinal tract (GIT) are synthesized from the proteins of pigs, but not reabsorbed protein (digestive enzyme, mucin protein, and serum albumin), sloughed intestinal cells, and bacterial protein by fermentation in the GIT [18,19]. Among the BEL of AA, Glu, Asp, Leu, and Thr were relatively greater contents [20]. However, in pigs fed N-free diets, Pro and Gly contents in ileal digesta were relatively high [1,19,21] that agreed with the present work. This indicates that pigs were not in a normal physiological state [20]. In pigs fed an N-free or AA-deficient diet, the protein synthesis is insufficient, and thus, a greater protein mobilization occurs and a greater amount of dispensable AA, especially in Pro and Gly, are excreted in the ileal digesta [19]. However, in the present work, the addition of an AA mixture at the adaptation period in pigs fed an N-free diet did not decrease the Pro and Gly contents in the ileal digesta. The reason is likely that the His, Leu, and Phe contents in the N-free diet with AA supplementation were not sufficient to prevent protein mobilization. Although the AA mixture was added to the N-free diet during the adaptation period, the Arg, His, Leu, and Phe contents in the N-free diet with AA addition group were less than 35% of the requirement of AA for growing pigs [1]. Therefore, the imbalance of AA composition in an N-free diet with the supplementation of AA may not affect the BEL of AA in the present work.

The AID and SID of CP and AA from pigs fed the diet containing corn and SBM without AA mixture addition were within a range of the previous studies [1,6]. However, some previous studies reported relatively less digestibility of AA in SBM [22]. The deviation is likely due to the inclusion rate of soybean hulls. Soybean hulls were relatively greater insoluble dietary fiber contents than SBM [23], which might reduce the SID of AA. The dietary fiber contents were less digestible in the GIT of pigs and inhibit the digestion of other nutrient compositions [24,25]. The SID of CP and AA also decreased as the dietary fiber contents increased that might explain the SID of most AA in corn was relatively less than that in SBM. In the present work, the AID of Met, Thr, and Glu in SBM was greater than that in corn, but not differ between the SID of Met, Thr, and Glu in corn and SBM. The reason is likely that a proportion of the BEL to undigested CP and AA in pigs fed a low-protein diet was greater than that in pigs fed a high-protein diet [3].

In the present work, to determine the effect of an AA addition during the adaptation period on the ileal digestibility of CP and AA in corn, the AA mixture was not provided during the collection period [7,9]. Although the crystalline AA was supplemented during the adaptation period, the crystalline AA may not be totally absorbed in the GIT of pigs but may lower AID and SID of AA [7,9]. The AID and SID of AA in corn with AA supplementation during adaptation period [7,9] were relatively less than the reported AID and SID of AA values in pigs whose diet contained only corn as a sole N source [1]. The reason is likely due to the lower body weight of the pigs. Weaning pigs have only relatively immature digestive capacity and relatively small intestine size that resulted in less ileal digestibility of AA [26]. Sauer et al [7] used weaning pigs with 5 kg of initial body weight that might reduce the ileal digestibility of AA. However, Pedersen et al [9] used growing pigs of 76 kg to determine the ileal digestibility values. Another possibility for the lower AID and SID of AA in corn is that a lower supplementation of chromic oxide concentrations (0.3%) was used in the experimental diets to determine the ileal digestibility [9]. In the previous studies, the Cr recovery was reported to be 71% to 85% at the end of the small intestine [27] that may have decreased the ileal digestibility of AA in corn [28]. The lower AA composition in corn also might increase the variation of AID and SID of AA [3]. However, in corn and SBM with AA supplementation, the AID and SID of CP and AA did not differ [10] that agreed with the present study. The SID of the AA mixture was also about 100% [29]. Taken together, the addition of AA did not affect ileal digestibility of most AA in corn and SBM.

The improved AID and SID of CP and Pro in the corn with AA supplementation at the adaptation period, but without increased AID and SID of CP and Pro in SBM were observed in this study. The reason for the only increase of AID and SID of CP and Pro in corn may be due to less Pro concentrations in ileal digesta in corn-based diet with AA supplementation group that are agreement with pigs fed corn distillers dried grains with solubles with casein diets [5]. In pigs fed an N-free diet, the high protein mobilization resulted in relatively greater dispensable AA especially in Pro and Gly contents in ileal digesta [19]. In other words, in pigs fed an AA-balanced diet, the Pro and Gly contents in ileal digesta were relatively less than in pigs fed an N-free diet. However, the BEL of CP and AA representing the secretion of enzymes did not differ in pigs fed the N-free diet regardless of AA mixture supplementation in the present work. The deviation may be due to the Arg, His, Leu, and Phe contents in corn. The Arg, Leu, and Phe contents in corn with AA supplementation were over 65% of the requirement of AA. The high contents of the essential AA (Arg, His, Leu, and Phe) in corn with the AA supplementation group might decrease protein mobilization that might result in less Pro and Gly contents in ileal digesta.

## CONCLUSION

The addition of amino acids to a low-protein experimental diet during the adaptation period does not affect the basal endogenous losses of amino acids and standardized ileal digestibility of indispensable amino acids in pigs.

## Figures and Tables

**Table 1:** Analyzed composition of corn and soybean meal (as-fed basis)

Item (%)	Corn	Soybean meal
Dry matter	87.69	88.86
Crude protein	10.13	43.52
Indispensable amino acids		
Arg	0.47	3.25
His	0.24	1.06
Ile	0.39	2.02
Leu	0.98	3.37
Lys	0.42	2.67
Met	0.32	0.66
Phe	0.42	2.25
Thr	0.43	1.81
Trp	0.09	0.54
Val	0.51	2.02
Dispensable amino acids		
Ala	0.60	1.99
Asp	0.75	5.20
Cys	0.43	0.87
Glu	1.76	8.03
Pro	0.80	2.29
Ser	0.43	2.26
Tyr	0.31	1.31
Gly	0.45	1.94

**Table 2:** Ingredient composition of experimental diets for adaptation period (as-fed basis)

Item (%)	Corn		Soybean meal		Nitrogen-free	
		
	-	Amino acid	-	Amino acid	-	Amino acid
Ground corn	87.05	87.05	-	-	-	-
Soybean meal (CP 45%)	-	-	25.00	25.00	-	-
Corn starch	7.25	-	49.50	48.65	70.60	62.60
Sucrose	-	-	20.00	20.00	20.00	20.00
Soybean oil	2.00	2.00	2.00	2.00	2.00	2.00
Cellulose	-	-	-	-	3.00	3.00
L-Lys·HCI (78.8%)	-	-	-	0.40	-	-
DL-Met (99%)	-	-	-	0.25	-	-
L-Thr (99%)	-	-	-	0.20	-	-
Dicalcium phosphate	1.60	1.60	1.60	1.60	2.00	2.00
Ground limestone	0.70	0.70	0.50	0.50	0.50	0.50
Potassium carbonate	-	-	-	-	0.40	0.40
Magnesium oxide	-	-	-	-	0.10	0.10
Sodium chloride	0.50	0.50	0.50	0.50	0.50	0.50
Vitamin-mineral mix^1)^	0.40	0.40	0.40	0.40	0.40	0.40
Chromic oxide	0.50	0.50	0.50	0.50	0.50	0.50
Amino acid mixture	-	7.25	-	-	-	8.00

1) Provided the following quantities per kg of complete diet: vitamin A, 8,000 IU; vitamin D_3_, 1,600 IU; vitamin E, 1 IU; vitamin K_3_, 0.4 mg; thiamin, 0.4 mg; riboflavin, 1.2 mg; pyridoxine, 0.8 mg; vitamin B_12_, 0.004 mg; pantothenic acid, 4.0 mg; folic acid, 0.8 mg; niacin, 8.0 mg; biotin, 0.08 mg; Cu, 1.0 mg as copper sulfate; Fe, 16 mg as iron sulfate; I, 1.0 mg as calcium iodate; Mn, 48 mg as manganese sulfate; Zn, 60 mg as zinc sulfate; Co, 0.4 mg as cobaltous carbonate; Mg, 8 mg as magnesium oxide; and choline chloride, 100 mg.

**Table 3:** Composition of the crystalline amino acid mixture (%, as-fed basis)

Item	Inclusion rate
L-Glycine (99%)	58.0
L-Lysine·HCl (78.8%)	16.3
DL-Methionine (99%)	3.8
L-Threonine (99%)	6.2
L-Tryptophan (99%)	2.2
L-Isoleucine (99%)	4.7
L-Valine (99%)	4.8
L-Histidine (99%)	1.1
L-Phenylalanine (99%)	2.9

**Table 4:** Daily experimental diet feeding procedures

Day	Corn		SBM		Nitrogen-free	
		
	-	AA	-	AA	-	AA
1	Corn	Corn+AA	SBM	SBM+AA	N-free	N-free+AA
2	Corn	Corn+AA	SBM	SBM+AA	N-free	N-free+AA
3	Corn	Corn+AA	SBM	SBM+AA	N-free	N-free+AA
4	Corn	Corn+AA	SBM	SBM+AA	N-free	N-free+AA
5 Morning	Corn	Corn+AA	SBM	SBM+AA	N-free	N-free+AA
5 Afternoon	Corn	Corn	SBM	SBM	N-free	N-free
6^1)^	Corn	Corn	SBM	SBM	N-free	N-free
7^1)^	Corn	Corn	SBM	SBM	N-free	N-free

AA, amino acid; SBM, soybean meal.

1) Ileal digesta samples were collected on days 6 and 7.

**Table 5:** Chemical composition of experimental diets (as-fed basis)

Item (%)	Corn		Soybean meal		N-free	
		
	-	Amino acid	-	Amino acid	-	Amino acid
Dry matter	88.50	88.72	92.41	92.42	91.94	93.35
Crude protein	9.06	16.51	11.41	12.11	0.25	8.16
Ether extract	3.71	4.57	2.40	1.90	1.14	1.71
Neutral detergent fiber	7.82	7.91	2.60	2.66	3.16	3.23
Acid detergent fiber	2.01	1.98	1.10	1.36	2.87	3.23
Ash	5.06	4.83	4.28	4.43	3.66	3.66
Calcium	0.88	0.91	0.82	0.81	0.76	0.82
Phosphorus	0.62	0.61	0.49	0.47	0.39	0.37
Indispensable amino acids						
Arg	0.43	0.40	0.77	0.86	0.01	0.00
His	0.21	0.43	0.28	0.31	0.00	0.11
Ile	0.35	0.64	0.54	0.51	0.00	0.38
Leu	0.87	0.90	0.89	0.83	0.01	0.02
Lys	0.38	1.29	0.69	0.99	0.01	0.96
Met	0.16	0.34	0.18	0.31	0.01	0.19
Phe	0.37	0.54	0.56	0.51	0.01	0.21
Thr	0.39	0.75	0.43	0.70	0.01	0.48
Trp	0.09	0.24	0.12	0.13	<0.01	0.14
Val	0.46	0.78	0.53	0.51	0.01	0.38
Dispensable amino acids						
Ala	0.54	0.53	0.51	0.50	0.01	0.01
Asp	0.69	0.68	1.40	1.31	0.01	0.00
Cys	0.26	0.24	0.22	0.22	0.01	0.01
Glu	1.68	1.65	2.11	1.93	0.02	0.01
Pro	0.71	0.66	0.61	0.56	0.01	0.00
Ser	0.41	0.39	0.57	0.58	0.01	0.19
Tyr	0.23	0.25	0.38	0.30	0.00	0.00
Gly	0.39	4.58	0.57	0.57	0.02	4.82

**Table 6:** Effects of an amino acid mixture during the adaptation period on apparent ileal digestibility of crude protein and amino acids in corn and soybean meal^1)^

Item (%)	Corn		Soybean meal		SEM	p-value^2)^		
		
	-	AA	-	AA		Ing	AA	Ing×AA
Crude protein	66.6	75.5	71.9	73.8	2.0	0.312	0.007	0.060
Indispensable AA								
Arg	66.7^b^	76.2^a^	79.5^a^	80.8^a^	2.2	<0.001	0.014	0.049
His	77.4	77.7	85.7	83.7	1.7	<0.001	0.521	0.414
Ile	78.5	79.7	86.1	85.8	1.1	<0.001	0.678	0.462
Leu	86.3	85.6	86.4	86.6	0.9	0.545	0.786	0.599
Lys	76.3	78.3	89.9	87.5	1.4	<0.001	0.833	0.082
Met	87.5	87.7	90.9	88.5	1.0	0.035	0.247	0.162
Phe	83.5	83.1	87.3	87.6	1.1	<0.001	0.939	0.703
Thr	70.1	70.5	74.7	72.9	1.9	0.038	0.631	0.469
Trp	72.4^b^	77.5^a^	80.6^a^	80.0^a^	1.0	<0.001	0.033	0.009
Val	78.8	79.8	82.9	82.2	1.2	0.009	0.903	0.420
Dispensable AA								
Ala	76.7	78.0	74.2	75.7	1.5	0.123	0.364	0.973
Asp	71.0	74.6	83.2	83.0	1.1	<0.001	0.140	0.095
Cys	79.8	82.3	80.3	76.9	1.5	0.135	0.781	0.070
Glu	85.1	84.9	87.1	87.4	0.8	0.015	0.951	0.692
Pro	–27.6	43.3	–76.8	–55.3	19.7	<0.001	0.013	0.151
Ser	66.7	70.1	76.3	75.9	1.4	<0.001	0.297	0.186
Tyr	77.6	77.6	88.1	87.8	1.7	<0.001	0.915	0.906
Gly	37.6	55.7	53.2	56.3	4.3	0.059	0.017	0.080

AA, amino acid; SEM, standard error of the means.

1) Each least square mean represents 6 observations except for the apparent ileal digestibility of Met in pigs fed corn without AA group and soybean meal with AA group (n = 5).

2) Ing, feed ingredient; AA, AA addition; Ing×AA, interaction between feed ingredient and AA addition.

a,b Least squares means within a row without common superscript differ (p<0.05).

**Table 7:** Effects of an amino acid mixture on basal ileal endogenous losses of crude protein and amino acids^1)^ (g/kg dry matter intake)

Item	-	AA	SEM	p-value
Crude protein	17.71	18.89	2.550	0.753
Indispensable AA				
Arg	0.89	0.92	0.161	0.873
His	0.18	0.18	0.027	0.856
Ile	0.24	0.25	0.031	0.923
Leu	0.36	0.38	0.039	0.715
Lys	0.29	0.28	0.040	0.767
Met	0.06	0.08	0.009	0.293
Phe	0.17	0.19	0.021	0.390
Thr	0.45	0.52	0.046	0.314
Trp	0.10	0.10	0.012	0.958
Val	0.31	0.36	0.027	0.200
Dispensable AA				
Ala	0.62	0.70	0.092	0.578
Asp	0.72	0.81	0.104	0.571
Cys	0.19	0.19	0.028	0.859
Glu	0.74	0.91	0.068	0.093
Pro	8.14	8.91	1.867	0.774
Ser	0.58	0.64	0.071	0.541
Tyr	0.12	0.16	1.297	0.973
Gly	1.95	1.88	0.277	0.857

AA, amino acid; SEM, standard error of the means.

1) Each least square mean represents 6 observations.

**Table 8:** Effects of an amino acid mixture during the adaptation period on standardized ileal digestibility of crude protein and amino acids in corn and soybean meal^1)^

Item (%)	Corn		Soybean		SEM	p-value^2)^		
		
	-	AA	-	AA		Ing	AA	Ing×AA
Crude protein	83.5	93.6	86.0	88.8	2.0	0.507	0.002	0.054
Indispensable AA								
Arg	70.4	77.8	80.2	81.5	3.1	0.020	0.116	0.258
His	78.7	78.2	85.9	83.9	1.8	0.001	0.460	0.644
Ile	79.7	80.0	86.3	85.9	1.3	<0.001	0.990	0.809
Leu	87.2	86.0	86.6	86.9	1.0	0.889	0.642	0.468
Lys	77.6	78.8	90.2	87.7	1.7	<0.001	0.681	0.248
Met	88.2	87.8	90.9	88.5	1.1	0.125	0.183	0.350
Phe	84.3	83.2	87.4	87.7	1.2	0.003	0.723	0.513
Thr	72.1	71.5	75.0	73.2	2.4	0.197	0.489	0.724
Trp	74.2	77.8	80.7	80.0	1.6	0.009	0.324	0.170
Val	80.0	80.1	83.1	82.4	1.4	0.053	0.825	0.734
Dispensable AA								
Ala	78.9	78.8	74.7	76.2	2.1	0.111	0.731	0.682
Asp	73.0	75.4	83.8	83.6	1.4	<0.001	0.453	0.394
Cys	81.0	82.4	80.5	77.1	1.7	0.092	0.574	0.161
Glu	86.4	85.8	87.6	88.1	1.0	0.077	0.959	0.534
Pro	–4.3	56.2	–70.4	–48.4	22.7	<0.001	0.033	0.291
Ser	69.2	70.8	76.7	76.3	2.0	0.003	0.751	0.611
Tyr	78.6	77.9	88.2	87.9	1.9	<0.001	0.755	0.899
Gly	46.7	58.6	54.7	57.8	6.5	0.542	0.219	0.458

AA, amino acid; SEM, standard error of the means.

1) Each least square mean represents 6 observations except for the standardized ileal digestibility of Met in pigs fed corn without AA group and soybean meal with AA group (n = 5).

2) Ing, feed ingredient; AA, AA addition; Ing×AA, interaction between feed ingredient and AA addition.
